# Effect of *Inonotus obliquus* polysaccharide on composition of the intestinal flora in mice with acute endometritis

**DOI:** 10.1371/journal.pone.0259570

**Published:** 2021-11-05

**Authors:** Binhong Hu, Yuqing Dong, Wenjing Zhou, Yichuan Ma, Luyao Li, Xianhua Fu, Wenxuan Zhang, Yuanyue Luo, Jingyu Pu, Xin Deng, Rong Zhang, Songqing Liu

**Affiliations:** 1 College of Chemistry and Life Sciences, Chengdu Normal University, Chengdu, China; 2 Sichuan Provincial Key Laboratory for Development and Utilization of Characteristic Horticultural Biological Resources, Chengdu Normal University, Chengdu, China; 3 College of Forestry, Sichuan Agricultural University, Chengdu, China; 4 School of Physical Science and Technology, Shanghai Tech University, Shanghai, China; University of Illinois Urbana-Champaign, UNITED STATES

## Abstract

*Inonotus obliquus* Polysaccharide (*IOP*) is a large molecule extracted from *Inonotus obliqus*, a medicinal fungus, which has a wide range of biological activities and has been shown to be associated with inflammation. The purpose of this study is to investigate whether IOP can help to reduce acute endometritis by regulating intestinal flora. We observed pathological changes in mice with endometritis following treatment with IOP and evaluated changes in the levels of interleukin-6 (IL-6), interleukin-1β (IL-1β) and tumor necrosis factor α (TNF-α), and further studied the effects of IOP on the intestinal flora of endometritis mice using 16S rRNA high-throughput sequencing. The results showed that IOP improved the condition of uterine tissues and reduced the release of pro-inflammatory cytokines. Meanwhile, the 16S rRNA sequencing results showed that IOP could regulate the changes in intestinal microflora at the level of genera, possibly by changing the relative abundance of some genera.

## Introduction

Endometritis is an infectious uterine disease that is closely related to infertility [1, 2]. According to histological criteria, endometritis can be divided into acute, chronic and fibrosis subtypes. The histological features of acute endometritis are congenital and intrahepatic polynuclear infiltration [1]. The cause of acute endometritis is bacterial infection [3], including *Escherichia coli*, *Staphylococcus aureus*, or *Streptococcus Lipopolysacchride* (LSP) in the cell wall of Gram-negative bacteria plays an important role in inflammation [4–6].

There are rich interactions between microorganisms and hosts [7]. Under normal conditions, large numbers of bacteria form a microbial barrier to protect the intestinal tract in order to maintain gastrointestinal stability and resist the invasion of pathogenic bacteria [8]. Generally speaking, the host and enteroviruses exist in a dynamic equilibrium, which if disrupted can lead to various diseases. There is growing evidence that disequilibrium of intestinal flora can contribute to the development of diabetes [9], joint inflammation [10], hepatitis [11], and neuroinflammation [12]. In addition, some studies have shown that an imbalance of enteroviruses can lead to increased estrogen [13], which is closely related to inflammation [14]. Therefore, the regulation of estrogen by intestinal flora may be related to the development of endometritis in the uterus.

Natural polysaccharides are associated with a wide range of biological effects and can provide therapeutic value by directly affecting metabolism *in vivo*, the polysaccharides concentrate symbiotic bacteria to form a biological barrier to protect the host from pathogens [15, 16]. In addition, they can change the composition of rose organisms such as *Glycyrrhiza Uralensis* Fisch Polysaccharides (*GCP*) that affect can inhibit tumor growth *in vivo* [17]. Pumpkin polysaccharide (*PP*) reduces the pathogenesis of type II diabetes in rats by regulating intestinal flora [18]. *Inonotus obliqus* is an edible and medicinal fungi that grows in frosty conditions in Asia and Europe and has been used as folk medicine in Russia to treat tumors and stomach ulcers. Furthermore, it has also been used to treat and prevent cancer, diabetes, cerebrovascular diseases and other diseases in Europe [19–21]. The polysaccharide components of *Inonotus obliqus* have an extensive range of biological characteristics such as the anti-inflammatory, anti-oxidative and anti-viral activity [22–24]. Previous research has found that *Inonotus obliquus* polysaccharide (*IOP*) may improve chronic pancreatitis (*CP*) in mice and promote the intestinal flora at the same time [25]. However, the potential role of IOP for the treatment of endometritis has not been studied, and the correlation between endometritis and intestinal flora has not been confirmed.

To investigate the relationship between endometritis and intestinal flora, pathological changes in mice with endometritis treated with IOP and the levels of IL-6, IL-1β and TNF-α were observed. We also performed an additional study to analyze the effect of IOP on intestinal flora in mice with endometritis using 16S rRNA high-throughput sequencing.

## Materials and methods

### Materials

*Inonotus obliqus* was provided by the Veterinary medicine laboratory of People’s Friendship University of Russia. Phosphate buffered saline (PBS) was purchased from Beijing Labgic Technology Co. (Beijing, China), and lipopolysacchride (LPS) was purchased from Sigmal-Aldrich (USA).

### The extraction of IOP

IOP was extracted according to traditional methods using hot water extraction followed by centrifugation [26]. Briefly, The IOP was crushed and the powder was degreased with petroleum ether. After degreasing, the residue is dried at low temperature. Heat the defatted residue and boil it with distilled water, repeat three times. The filtrates were combined, the solvent was recovered by reduced pressure, and 1% trichloroacetic acid was added to precipitate the protein. After centrifugation, the filtrate is concentrated into a liquid extract form. After precipitation with absolute ethanol, it was placed at 0°C overnight. The alcohol precipitation solution is centrifuged by a high-speed centrifuge to obtain a crude polysaccharide precipitate. The precipitate is washed 2–3 times with a small amount of absolute ethanol to obtain a crude polysaccharide product.

### Experimental processing

The animal study protocol was approved by the Animal Care Office of Chengdu Normal University, Chengdu, China and complied with the ARRIVE guidelines and followed the National Institutes of Health guide for the care and use of Laboratory animals.

SPF BALB/C female mice were purchased from Dossy Experimental Animals Co. (Chengdu, China). The mice were housed in a settled environment (temperature: 25±3°C, humidity: 75±5%) with adequate food and water. The mice were exposed to light for 12h each day. The mice were randomly divided into four groups with twelve mice in: Control group; LPS group; LPS+IOP group. To avoid infection by other bacteria, mice were pretreated with streptomycin [27]. A murine model of LPS-induced endometritis was established as previously reported [28]. LPS group injected with 20ul LPS (3mg/ml) in vivo [29, 30]. The control group were injected with the same amount of PBS, After 3 h, the LPS+IOP group were given IOP orally (150 mg/kg) [30]. The mice were monitored every hour for temperature, vaginal bleeding or death. During this process, none of the mice exhibited morbidity. After 9 h, the mice were euthanized, and uterine and colorectal tissues were collected and stored at -80°C.

### Histopathology analysis

Uterine tissue was fixed by paraformaldehyde followed by trimming, dehydration, and embedding in paraffin. The 5 μm thick slices were dyed using hematoxylin and eosin (H&E) before visualization under a microscope (Nikon, Eclipse Ci-L, Japan).

### Inflammatory cytokine detection

The expressions of interleukin (IL)-6, IL-1β and TNF-α were detected by quantitative real-time polymerase chain reaction (PCR). According to the manufacturer’s instructions, total RNA was extracted from the tissue of the uterus using MiniBEST Universal RNA Extraction Kit (Takara, Japan) and reverse transcribed into cDNA. The cDNA product was diluted with Fast qPCR Master Mix (High Rox, BBI, ABI) on a StepOne Plus fluorescent quantitative PCR instrument (ABI, Foster, CA, USA). Primers (Table 1) were designed using Primer Premier 5.0 software, and relative quantification of target gene expression was performed using the 2^-ΔΔCt^ method.

**Table 1:** Primers for qPCR.

Name	Primer sequence	Product size(bp)
IL-6	Forward: TCTTGGGACTGATGCTGGTG	132
	Reverse: CATGTGTAATTAAGCCTCCGACT	
IL-1β	Forward: GTAATGAAAGACGGCACACCC	181
	Reverse: CAGGCTTGTGCTCTGCTTGTG	
TNF-α	Forward: TGTCTCAGCCTCTTCTCATTCC	152
	Reverse: TTTGTGAGTGTGAGGGTCTGG	
GAPDH	Forward: GGTTGTCTCCTGCGACTTCA	183
	Reverse: TGGTCCAGGGTTTCTTACTCC	

### DNA extraction and library construction

Total genomic DNA from colorectal tissue was extracted using QIAamp 9 PowerFecal QIAcube HT kit (QIAGEN, 51531) according to the manufacturer’s instructions. Concentration of DNA was verified using a NanoDrop (Thermo Fisher, 2000) and agarose gel electrophoresis. The genomic DNA was used as template for PCR amplification with the barcoded primers and Tks Gflex DNAPolymerase (Takara, R060B). According to bacterial diversity analysis, V3-V4 variable regions of 16S rRNA genes was amplified with universal primers 343 F and 798 R (343F: 5’- TACGGRAGGCAGCAG -3’,798R: 5’-AGGGTATCTAATCCT -3’ [31]). The quality of amplifiers was confirmed by gel electrophoresis, purified by AMPure XP bead (Agencourt), followed by another round of PCR amplification. After purification of the AMPure XP bead, Qubit dsDNA analysis kit (Life Technologies, Q32854) was used to quantify the final amplitor. An equal number of purified amplicon were pooled for subsequent sequencing.

### Bioinformatics analysis

Paired end—reads were preprocessed with Trimmomatic software [32], pruned and assembled with FLASH software after trimming [33]. The assembly parameters were as follows: minimum overlap 10 bp, maximum overlap 200 bp, and maximum error ratio 20%. Homologous sequences or those less than 200 bp were abandoned. In total, 75% of base readings above Q20 were retained. Then, the readings with the chimera were detected and removed. These steps were implemented using QIIME software (version 1.8.0) [34]. Vsearch software was used to generate operational taxonomic units (OTU) by removing primer sequences and clustering with a cut-off value of 97% similarity [35]. A representative reading for each OTU was selected using the QIIME package. All representative reads were annotate in line with the Silva database version 123 using the RDP classifier (confidence threshold was 70%) [36].

### Statistical analysis

Statistical analyses were performed using GraphPad Prism 8 (GraphPad InStat Software, USA). Comparison between groups was performed using one-way ANOVA, and data were expressed as mean±SEM. P<0.05 was considered statistically significant.

## Results

### IOP influences LPS-induced uterine inflammation

**Fig 1:**
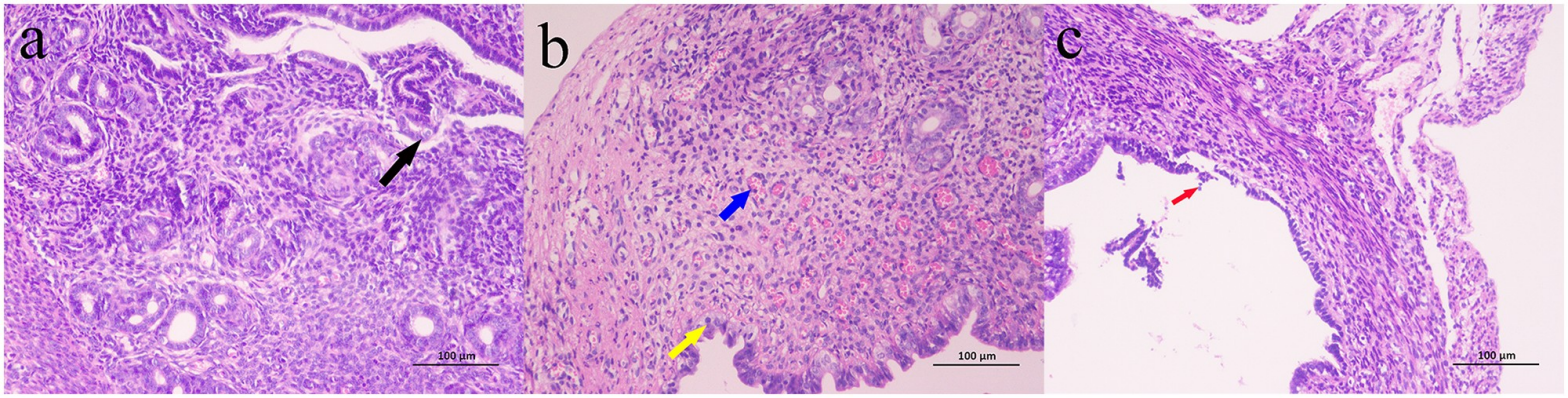
Effect of IOP on the histopathology of endometritis. (a). Control group. Local exfoliation of endometrial epithelial cells (black arrow). (b). LPS group. A small number of endometrial and glandular epithelial cells were swollen, the cytoplasm was loose and light stained (yellow arrow), and the lamina propria was heavily congested and dilated (blue arrow). (c). LPS+IOP Group. Local endometrial epithelium narrowed and a few endometrial epithelial cells shed (red arrow). (Hematoxylin and eosin staining, magnification 200 ×).

**Fig 2:**
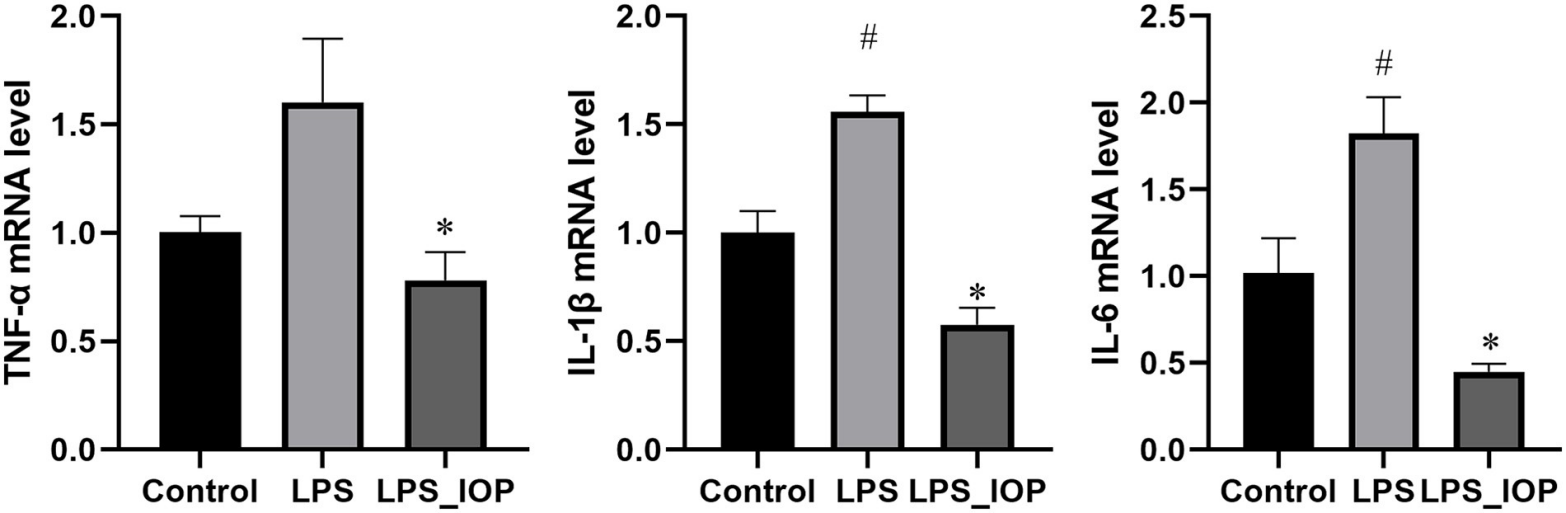
Effects of IOP on inflammatory cytokines TNF-α, IL-6, IL-1β. Mean ±SD was used for data processing. Three replicates were processed in each group. ^#^*P* < 0.05 vs. Control group, *P < 0.05vs. LPS group.

Compared with the control group, the lamina propria of the uterine tissues under LPS induction were swollen with a large number of capillary congestion and enhanced with eosinophils which improved after IOP treatment (Fig 1). Meanwhile, the levels of IL-6, IL-1β and TNF-α in uterine tissue were increased after LPS induction and decreased after IOP treatment (Fig 2).

### The total structure of the gut microbiota

**Fig 3:**
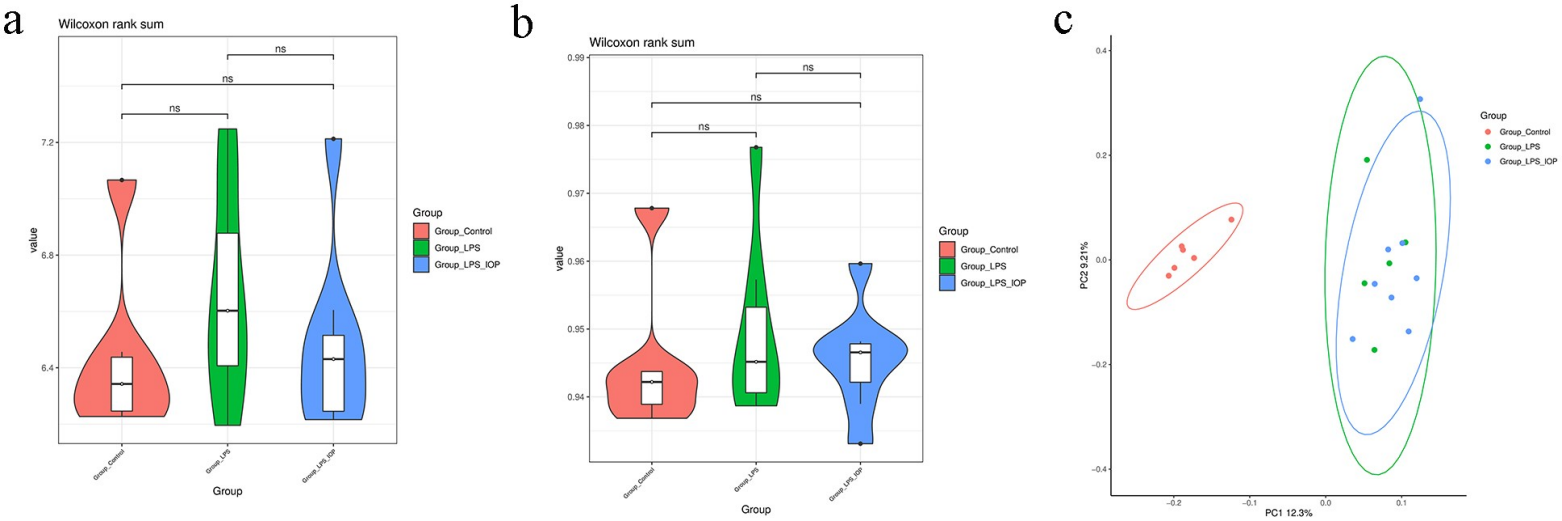
The total structure of the gut microbiota. (a). Shannon index of α diversity analysis. (b). Simpson index of α diversity analysis, ns means no difference. (c). Principal Co -ordinates analysis (*PCoA*) of β diversity.

After quality control of the original data obtained from high-throughput sequencing, the Clean Tags were distributed between 85767 and 93865 The Clean Tags obtained through the removal of chimera relative to the valid Tag data were distributed between 78943 and 86592 and the final total OUT number was 6143 (S1 Table). The diversity of each group was analyzed by α diversity test; Shannon and Simpson indexes were measured by Wilcoxon Rank SUM test. The results (Fig 3a and 3b) showed that there was no significant difference in α diversity index between the groups. Simultaneously, we used β diversity analysis to compare the difference between the group samples. According to PCoA two-dimensional chart (Fig 3c), in mice with LPS-induced endometritis, a significant change in the microbial community was observed. However, there was not obviously altered in the microbiome after IOP treatment.

### Changes in gut microbiota composition

**Fig 4:**
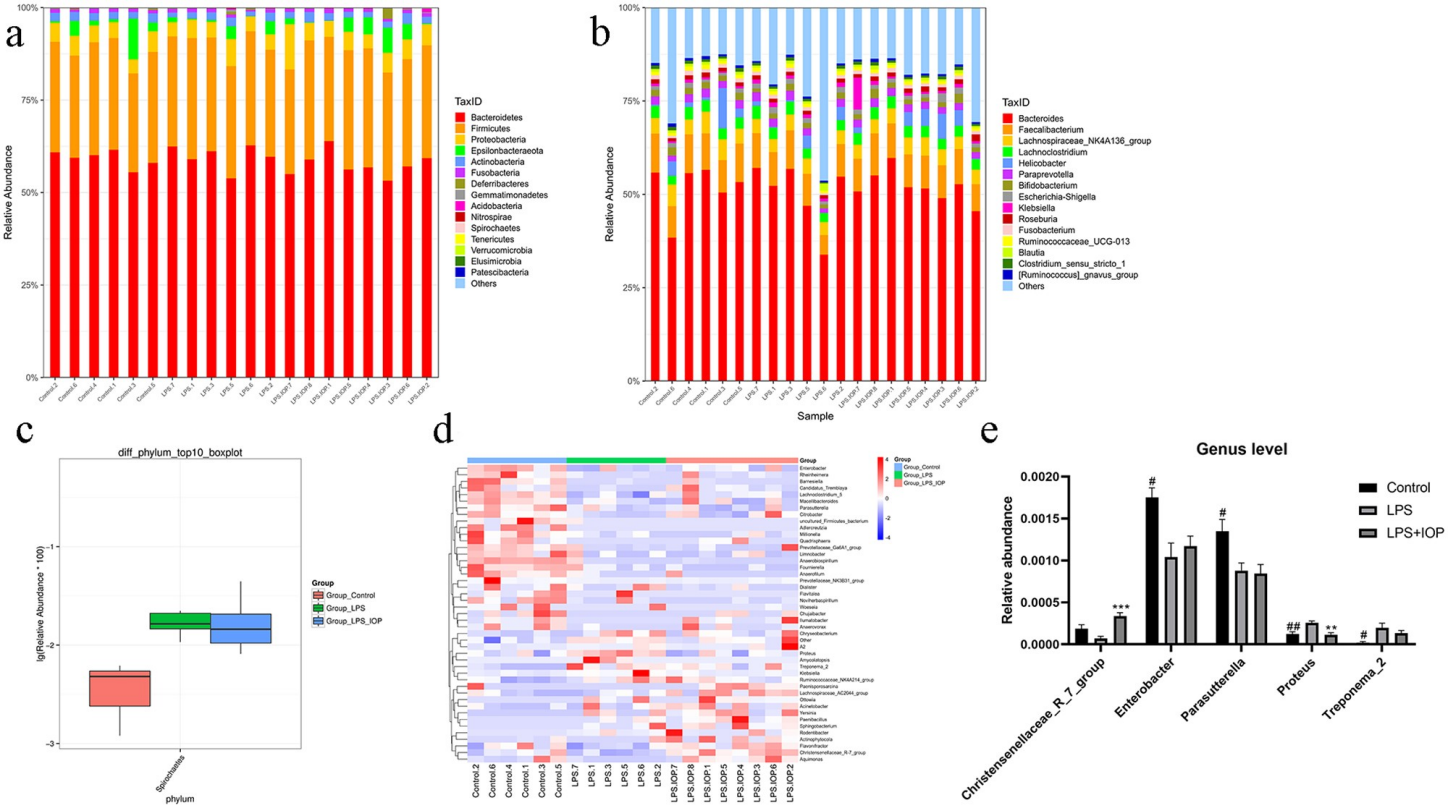
Changes in gut microbiota composition. (a). Species relative abundance of species at the phylum level. (b). Species relative abundance of species at the genus level. (c). Boxplot representing differences at the phylum level. (d). Heat map of differences at the genus level. Red indicates relatively high species abundance and blue indicates relatively low species abundance. (e). Significant species differences among the control group, LPS group and IOP interference group. ^#^*P* < 0.05, ^##^*P* < 0.01,vs. Control group, **P < 0.01, ***P < 0.001vs. LPS group.

After obtaining the OTU annotation results where multiple OTUs corresponded to the same genus or species, the classification results were summarized to obtain the relative abundance of samples at each level (S2 Table). Fig 4a and 4b shows the relative abundance of TOP15 species in each group. The *Bacteroidetes*, *Firmicutes*, and *Proteobacteria* are grouped at the level of Phylum, whereas *Bacteroides*, *Faecalibacterium*, *Lachnoclostridium*, *Helicobacter*, *Paraprevotella*, *Bifidobacterium* were grouped at the genus level. We also analyzed the different species of each group using a Kruskal Wallis algorithm (Fig 4c and 4d). At the phylum level, *Spirochaetes* was found to be differentially expressed, while species at the genus level included *Klebsiella*, *Lachnoclostridium_5*, *Enterobacter*, *Flavonifractor*, *Parasutterella*, *Treponema_2*, and *Christensenellaceae_R−7_group*. Fig 4e shows the difference microbiota of each group at the genus level, compared to the Control group, *Enterobacter* (P<0.05) and *Parasutterella* (P<0.05) were significantly decreased in LPS group, while *Proteus* (P<0.01) and *Treponema_2* (P<0.05) were increased, after IOP intervention, *Christensenellaceae_R−7_group* (P<0.001) was significantly increased, while *Proteus* (P<0.01) was decreased.

## Discussion

Endometritis is a bacterial uterine disease that occurs in women and female animals such as cows and sows, affecting quality of life in women and also modern agricultural production [2, 37]. LPS causes inflammation by inducing the production of pro-inflammatory cytokines such as IL-6, IL-1β and TNF-α [38]. Our results showed that mouse uterine epithelial cells were swollen and eosinophilic after LPS induction which was significantly improved by IOP treatment. At the same time, the levels of IL-6, IL-1β and TNF-α were increased after treatment with LPS and decreased by IOP. This indicates that IOP may ameliorate the symptoms of endometritis induced by LPS.

There is growing evidence that the diversity of gut microbes is linked to human health [39–41]. Previous studies have shown that the composition of the intestinal microflora has changed due to the induction of false evidence in endometrium and endometrial cancer [42], and the occurrence of endometritis is also related to intestinal microorganisms. To this end, we performed diversity analysis using 16S rRNA sequencing and found no clear effect of endometritis on the diversity of intestinal microflora. However, we did find differences between species of *Spirochaetes* at the phylum level, and in *Christensenellaceae_R*.*7_group*, *Parasutterella*, *Enterobacter*,*Treponema_2* and *Proteus* at the genus level in the composition of the gut microbiota.

*Spirochaetes* bacteria may be pathogenic [43], and we noted that the relative abundance of this species increased under the induction of LPS, while the abundance decreased after treatment with IOP, but not significantly. It indicated an increase in pathogenic bacteria in mice induced by LPS, whereas IOP intervention has little effect on bacteria in this phylum.

*Christensenellaceae_R*.*7_group* is a widespread human and animal microbe that been linked to conditions including obesity and inflammatory bowel disease [44], *Christensenellaceae_R*.*7_group* is not present in large amounts in obese people [39]; however, obese people have a higher risk of endometritis compared with more limited individuals [45]. LPS reduced the abundance of *Christensenellaceae_R*.*7_group*, while IOP significantly increased its abundance, even exceeding the normal level. This suggests that IOP may reduce LPS-induced endometritis in mice by increasing the abundance of *Christensenellaceae_R*.*7_group*.

*Parasutterella* has been shown to be an important microorganism that maintains gastrointestinal health in humans. Inflammatory bowel disease, obesity, diabetes, and fatty liver have been shown to be associated with the relative abundance of the species [46–49]. The relative abundance of *Parasutterella* was decreased after treatment with LPS and IOP. Although IOP intervention alleviates the symptoms of endometritis, it may not have an effect on *Parasutterella* and may even further reduce its relative abundance. In addition, *Parasutterella* has also been shown to be related to the homeostasis of bile acids [46] and the pathogenesis of cervicitis is related to the biosynthesis of primary bile acids. Not only that, cervicitis can cause a range of diseases including endometritis [50], which is consistent with the changes in relative abundance of *Parasutterella* observed in mice with endometritis.

In the previous uterine microbiota detection in patients with endometritis, fewer *Enterobacter* have been observed and it is speculated that the reduction in the number of *Enterobacter* may be related to the overgrowth of endometrial tissue [51], which is similar to the results observed in the intestine. In addition, as a pathogen [52], *Treponema_2* was increased after LPS induction, suggesting a role in inflammation. *Proteus* is a common symbiont of intestinal microbiome, which is widely considered to be pathogenic and also one of the pathogenic bacteria of endometritis [53]. The interference of IOP significantly reduced the abundance of *Proteus*, which also showed the function of IOP in alleviating endometritis. Interestingly enough, the effect of IOP on the gut microbiota is not obvious. The relationship between endometritis and the composition of intestinal flora and how the intestinal flora utilizes IOP under inflammation need further research. In summary, we further elucidated the role of IOP in endometritis, further analyzed the possible mechanism of action by analyzing the composition of intestinal microflora and identified the changes in the composition of microbial structure that may contribute to endometritis pathogenesis. For the specific role of changing microbiome in this is yet to be explained.

## Supporting information

S1 Table Raw data processing statistics. (TXT)

S2 Table Relative abundance of microbiota at phylum and genus levels. (TXT)
